# Exploring the Factors Underlying the Narrowing Urban Advantage in Child Mortality in Sub-Saharan Africa: A Scoping Review

**DOI:** 10.1007/s11524-025-00989-6

**Published:** 2025-09-04

**Authors:** Elle Quirey Parker, Gonnie Klabbers

**Affiliations:** 1 Queen’s University Belfast, Belfast, UK; 2 Maastricht University, Maastricht, Netherlands

**Keywords:** Urban advantage, Urban health, Urban slum, Urban population, Rural health, Urbanrural, Rural–urban, Health status disparities, Child mortality, Under 5 mortality, U5MR, Infant mortality, Child health, Sub-Saharan Africa, SSA

## Abstract

It is widely acknowledged that child mortality rates have been higher in rural than urban areas in sub-Saharan Africa (SSA); a phenomenon appreciated as the urban advantage. However, since at least the 1980s, this urban advantage has been narrowing, and in some cases reversing across SSA. While existing studies have primarily focused on establishing this relationship, few clearly define what constitutes urban or rural, with authors using different operationalizations. Even fewer explore the underlying drivers of change. Rural and urban health outcomes are associated with both the social determinants of health and the wider political economy of health systems. This study aims to elucidate the factors underpinning the narrowing urban advantage in by examining how such factors are differentially distributed and operate across urban and rural contexts. A scoping search was conducted for English-language peer-reviewed published articles after 1990 on urban and rural child health disparities in SSA. Databases used included PubMed, Embase, and Web of Science. Overall, 21 articles were included in the scope of this review. This review adhered to PRISMA-ScR guidelines (Preferred Reporting Items for Systematic Reviews and Meta-analyses Extension for Scoping Reviews). This review examines the extent to which urban areas in SSA continue to confer a survival advantage in child mortality, and the mechanisms underlying shifts in this trend. Four key categories of determinants—environmental, healthcare-related, sociodemographic, and disease/morbidity-related—consistently emerge across the literature, though their significance and strength vary across rural and urban settings. Notably, the review identifies a growing influence of intra-urban inequality, driven by informal urbanization and the expansion of slums, as a central factor in the narrowing urban advantage. The operationalization of urbanicity and rurality was inconsistent across studies, and rigid geographical classifications often obscured important spatial and contextual nuances. These findings underscore the limitations of conventional rural–urban comparisons and highlight the need for more nuanced frameworks that reflect the complex, evolving landscape of urban poverty and child health in SSA. The spatial reconfiguration of urban poverty appears to be modifying the distribution of child health risks in manners not captured by traditional urban–rural comparisons. Future research should focus on employing an urban continuum in demographic research, accounting for intra-urban inequities within the context of rapid urbanization processes which are altering the urban health landscape, and reshaping the social determinants of child mortality across the urban–rural spectrum.

**Supplementary Information:**

The online version contains supplementary material available at 10.1007/s11524-025-00989-6.

## Introduction

**Fig. 1:**
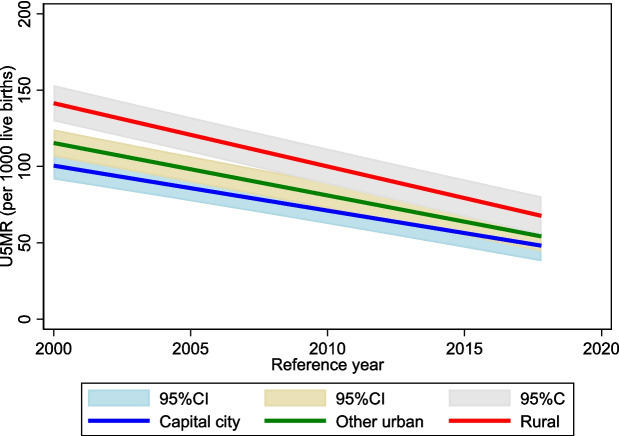
Trends in under 5 mortality across capital cities, urban, and rural areas in SSA. Extracted from Amouzou and colleagues [6]

While under-5 mortality rates (URMR) have declined across sub-Saharan Africa (SSA), the region still bears the highest global burden, with an U5MR of 75.8 per 1000 live births [1]. Most of these deaths occur during the neonatal period, with the proportion of U5MR having risen from 40 to 47% between 1990 to 2018 [2]. Since the 1980 s, urban areas in SSA have experienced lower U5MRs than their rural counterparts, attributable to better socioeconomic profiles, living standards and health infrastructure [3–5]. Waning rates of decline between urban and rural areas in SSA over the past three decades have resulted in a narrowed urban advantage (Fig. 1) and in some countries have reversed, demonstrating an urban penalty. These trends must be interpreted with nuance, due to inconsistent urban–rural definitions, and limited disaggregation of intra-urban differentials, rendering it difficult to assess and compare child mortality risk across and within countries. These classifications are not just academic, they are closely tied to different risk factors for under-five mortality. Where a child lives (e.g., formal urban area, informal settlement, rural village) shapes their exposure to key health determinants, such as sanitation, housing quality, and access to healthcare. These contextual differences underscore the need for more granular, context-specific analysis, as broad urban–rural comparisons can obscure important intra-urban inequalities, driving child health outcomes.

Tanzania displays the most pronounced urban penalty—urban neonatal mortality now doubles rural rates due to stagnant urban NMRs coupled with declining rural NMRs [7, 8]. Similar trends exist in Uganda, Ghana, and Kenya, where neonatal mortality disparities are most marked in the first week of life, most significantly linked to intrapartum care quality [8]. Guinea and Niger remain exceptions, with significantly higher rural child mortality rates [8]. For instance, in Tanzania, urban births are three times likelier to occur in hospitals, offering better intrapartum care access and therefore improving child survival [9]. However, this advantage is increasingly offset by rapid urban population growth, which strains finite healthcare resources, reduces care quality and availability, and contributes to the emerging urban penalty [9, 10].

Rapid urbanization is reshaping the distribution of health risks across SSA. The urban population is projected to rise from 25% of the total population in 1990 to nearly 60% by 2050 [11]. This exponential urban expansion paired with limited urban planning has led to overcrowding and the proliferation of slums and informal settlements [6]. These environments, marked by poor sanitation, unsafe housing, high infectious disease burden and limited access to health services are consistently associated with higher U5MR [12]. Average urban outcomes often mask substantial intra-urban disparities in U5MR, which can exceed urban–rural differences [13]. Disaggregating these patterns is vital to monitoring progress toward the Sustainable Development Goals (SDGs), particularly SDG 3 (Good Health and Well-being) and SDG 11 (Sustainable Cities and Communities), to reduce inequalities informing more effective, targeted and equitable interventions [14].

Understanding the urban penalty in SSA requires examining the complex urban–rural continuum often overlooked in research, where urban areas are typically treated as homogeneous. This simplification obscures distinctions among peri-urban, suburban, informal, and slum settlements due to varying definitions, unstandardized criteria, and political influences on urban classification [4, 8]. For instance, Ghana defines urban areas as regions with 5000 + residents, while Nigeria sets the threshold at 20,000 [15]. Such inconsistencies, coupled with wide variations in structural and social determinants across contexts, may obscure significant intra-urban disparities and hinders meaningful comparisons in U5MR trends. This review approaches urbanicity as heterogenous and continuous, seeking to better understand the drivers of the narrowing urban advantage and identify mechanisms behind spatial inequalities in child survival. To guide this analysis, the review explores two core research questions: Firstly, how are urban and rural settings operationalized in SSA, and secondly: What social determinants related to urbanicity, and rurality explain child mortality in these settings?

## Methods

### Search Strategy

A scoping search was conducted for English-language, peer-reviewed articles post-1990 on urban and rural child health disparities in SSA. The search covered PubMed, Embase, and Web of Science, with search terms comprising setting (urban/rural), child mortality, SSA, and synonyms. The complete search string consisting of all free text terms, Medical Subject Headings and Boolean operators were developed in conjunction with an information specialist and are outlined in Appendix A in supplementary material. Narrow terms were exploded to increase the yield of the search. Reference lists of relevant studies were also reviewed. The search was completed in April 2025.

### Eligibility Criteria

A total of 753 published studies discussing urban–rural child mortality in SSA were considered for inclusion in this review. EndNote software was used to manage titles and abstracts of these records. Studies were screened according to predetermined inclusion and exclusion criteria (Table 1).

**Table 1:** Inclusion and exclusion criteria

Inclusion	Exclusion
• Children under the age of 5, including neonates, residing in SSA	• Children aged 6 and over • Adolescents • African migrants not residing in Africa
• All geographically documented areas of SSA • Studies that stratified or disaggregated results by settlement type (e.g., urban formal vs informal, peri-urban vs rural) were included where possible	• Studies with no mention of urban/rural setting • Studies that did not include a rural–urban comparison or only reported data from a single setting (e.g., urban-only or rural-only)
• U5MR (defined as the probability a child dies before reaching exactly the age of 5 years, expressed per 1000 live births [16]) • Neonatal mortality rate (NMR) • Infant mortality rate (IMR)	• Child mortality rate including children aged 6 and over
• Peer-reviewed literature published in 1990 or later • Observational studies • Qualitative studies	• Grey literature • Publications before 1990 • Papers not in English • RCTs/trials/research protocols

### Data Collection and Analysis

Following exclusion processes, data from eligible studies were systematically extracted and charted to identify key themes and patterns. The collected data included author(s), year of publication, study design, country, factors used to construct urbanicity or/and rurality, and child mortality/morbidity outcomes. This approach facilitated the identification of differences which exist not only between urban and rural populations, respectively, but also those which exist among these groups. This permitted the identification of significant factors which comprised urbanicity and rurality respectively, analyzed according to their effect on child mortality. This review adhered to PRISMA-ScR guidelines (Appendix B in supplementary material).

## Results

**Fig. 2:**
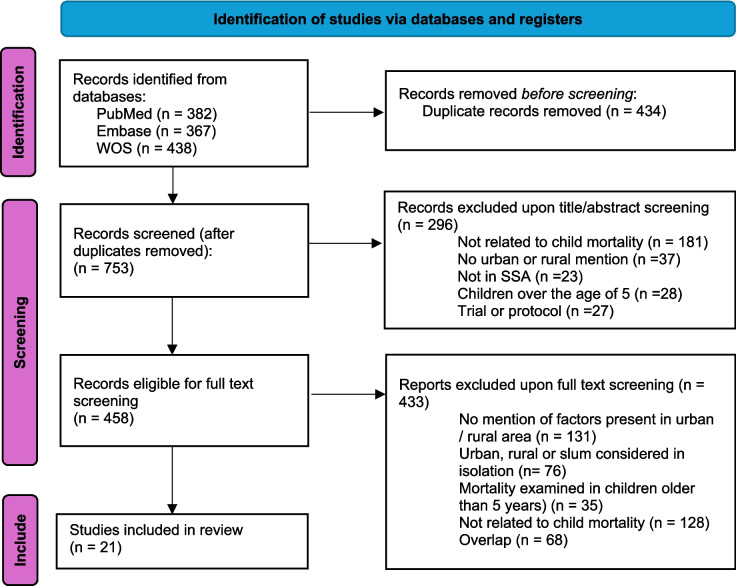
PRISMA flow diagram outlining the process of data identification, screening, and selection according to inclusion and exclusion criteria

After reviewing titles and abstracts, 458 articles were eligible for potential inclusion. Upon full-text screening, articles which were not related to child mortality; included children over 5 years old; were not in SSA; or did not describe urban or rural settings, were excluded. The final search output included 21 articles. The process of data collection and selection is illustrated Fig. 2.

This review encompassed 37 SSA countries: Angola, Cameroon, Benin, Congo Democratic Republic, Burkina Faso, Burundi, Eritrea, Chad, Comoros, Nigeria, Congo, Cote d’Ivoire, Eswatini, Ethiopia, Gabon, Gambia, Liberia, Ghana, Togo, Uganda, Guinea, Kenya, Lesotho, Madagascar, Morocco, Mozambique, Namibia, Malawi, Mali, Niger, Nigeria, Rwanda, Senegal, Sierra Leone, Sao Tome and Principe, South Africa, Tanzania, Zambia, Zimbabwe. Characteristics of these papers are summarized in Table 2.

**Table 2:** Characteristics of papers included in this review

Paper characteristic		Number of papers
Geographic scope	Multi country	10
	Country specific	11 total (Tanzania: 4) (Kenya: 3) (Nigeria: 2) (Ghana: 1) (Malawi: 1)
Outcome of interest	U5MR	10
	Child morbidity indicators (including access and use of child health services, stunting/malnutrition, immunization coverage, prevalence of acute respiratory infection (ARI) and diarrhea, Maternal health indicators (antenatal care ANC, presence of skilled birth attendance SBA, and caesarean delivery rates)	10
	Both child mortality (U5MR) and morbidity indicators	1
Urbanicity operationalization	Urban–rural dichotomy	12
	Urbanicity as continuous	3
	Stratified into urban formal and informal and rural	6
Data sources	DHS exclusively (1992–2021)	11
	DHS + other nationally representative household surveys (including Household budget surveys, population and housing census, Multiple Indicator Cluster Surveys, Urban Health and Demographic Surveillance System surveys, AIDS Indicator Survey (AIS), Malaria Indicator survey (MIS)	7
	DHS + satellite imagery (spatially classifying DHS clusters along urban continuum) OR Geographic Information System (GIS) data (travel time to cities) OR DHS + Global Human Settlement (GHS Layer data). Includes GHS-Settlement Model Grid, GHS-Urban Centre Database and GHS-Functional Urban Areas	3
Study Design	Cross-sectional	20
	Longitudinal	1
Research method	Qualitative	0
	Quantitative	21
Publication date	1990–2000	0
	2000–2010	2
	2010–2020	9
	2020–2025	10

### Operationalization of Urbanicity and Rurality

This review stratified findings based on how urbanicity was operationalized in the included studies, as summarized in appendix C in supplementary material. Three categories emerged: (1) binary urban–rural classification (appendix Ci in supplementary material), (2) stratified urban areas (formal, informal/slum, rural) (appendix Cii in supplementary material)), and (3) continuous urbanicity using spatial metrics or satellite imagery (appendix Ciii in supplementary material).

#### Urbanicity as a Binary Dichotomy

Studies using a dichotomous urban–rural framework reveal substantial inconsistencies, with no clear urban advantage or penalty. In Tanzania, neonates in urban areas faced higher mortality risks than rural counterparts (OR = 1.94; *p* = 0.006) despite greater service coverage, suggesting a growing urban penalty [8]. Conversely, across 35 SSA countries, pro-rural inequalities in U5MR were observed in 16 countries, pro-urban in 2, and no significant difference in 17 others [5]. In Malawi and Tanzania, urban–rural differences fluctuated, with declining urban advantage or reversal to urban penalty across morbidity indicators [7, 18]. Across central Africa, nearly all rural–urban inequity in U5MR was explained by differences in covariate distribution rather than effects [19]. These contradictions highlight the limitations of the urban–rural dichotomy, masking underlying intra-urban disparities and dynamic nature of health determinants.

#### Urbanicity as Stratified (Formal, Informal/Slum, Rural)

Stratified analyses reveal that children in informal urban settings consistently fare worse than their formal urban counterparts. While comparing urban informal settings to rural areas yielded mixed results, intra-urban inequities consistently exceeded urban–rural differences. In Kenya, urban non-slum (OR: 1.12, 95% CI 1.08–1.16) and slum areas (OR: 1.49, 95% CI 1.41–1.57) had higher child mortality than rural areas [20]. Fotso and colleagues demonstrated faster declines in U5MR in Kenya (rural = 22%, urban = 14%, slum = 39%) [21]. Kimani-Murage and colleagues explain the reversing urban penalty in Kenya through a narrowing urban–rural gap, with a more significant decline in rural areas [12]. Despite this, U5MR in urban slums remain highest (104/1000) relative to rural (72.5/1000) and non-slum urban (73/1000) areas [12]. Gunther and Harttgen found that in Ethiopia considering urban populations as homogenous masks large intra-urban inequalities [13]. Child mortality rates in urban slums were almost three times higher (125.9/1000) compared to formal urban areas (51.1/1000) in Ethiopia [13], with intra-urban differences exceeding urban–rural differences; U5MR was 65% higher in urban slums than formal urban areas, while rural U5MR is only 16% higher than formal urban areas. However, rural child mortality was higher than in urban areas overall, even when adjusting for urban slums. This indicates that while slums pose a higher health risk, urban access to basic infrastructure is still generally better than rural areas.

#### Urbanicity as a Continuous Concept

Studies operationalizing urbanicity as continuous leveraged composite indices, spatial metrics or satellite-derived spatial classifications​ to capture gradients in development and infrastructure. These approaches reveal that the relationship between urbanicity and child health is neither linear not uniformly protective. Across 26 SSA countries, urban children were 10% less likely to die than rural (OR = 0.9; CI 0.87–0.94), but this advantage varied by settlement type: compared to rural villages, mortality was lower in rural towns (OR = 0.94), urban clusters with deprivation (OR = 0.91), and urban centers with deprivation (OR = 0.84). However, children in deprived urban areas still face high health burdens, with only a weak mortality advantage (OR = 0.94; CI 0.88–1.01), while non-deprived urban clusters (OR = 0.85) and centers (OR = 0.83) show better outcomes [22]. In Tanzania, core urban areas exhibited higher NMRs (39.8/1000 live births) compared to peri-urban (24.8/1000 live births) and rural (21.9/1000) zones [4]. This highlights that urban advantage is often diluted or reversed in settings with infrastructural deficits or socioeconomic deprivation, suggesting that urbanicity alone does not ensure better child health. Instead, spatial gradients in service access and living conditions may more accurately explain health disparities across the urban continuum.

### Determinants Associated with Child Mortality in Urban and Rural Settings

The construction of rurality and urbanicity were inconsistent across all studies, with no clear cross-country nor urban–rural uniformity in factors underlying child mortality. Overall, the determinants were similar but varied in strength across papers in rural and urban areas. Based on the findings of included studies, four categories emerged: environmental, healthcare, sociodemographic, and disease and morbidity related. Appendix summarizes the findings across each of these four dimensions and their effect on the urban–rural gap.

#### Environmental Factors

Access to basic amenities like electricity, clean water, and sanitation is a significant marker of urbanicity. In West SSA, urban areas have greater access to these amenities (70%) than rural areas (20%), with these amenities and U5MR declining as city size decreased (*p* < 0.01) [23]. Having significantly reduced the probability of infant death (*β*: 0.13, *p* < 0.01) [19]. Over 70% of urban households in Kenya, both formal and informal, had improved water and sanitation, compared to only half of rural households [20]. However, Fotso and colleagues noted that rapid urban population growth is negatively correlated with access to safe drinking water (*r* = − 0.42; *p* = 0.07), increasing child mortality [21]. Across 22 SSA countries, those with most improved safe drinking water in urban areas had largest declines in U5MR, emphasizing the protective effect of amenities.

Housing quality also plays a critical role in child mortality related to urbanicity. There are higher risks of infant death in both urban non-slum (OR: 1.12, 95% CI: 1.08–1.16) and slum areas (OR: 1.49, 95% CI: 1.41–1.57) compared to rural areas. Improved housing was generally protective, especially in rural areas, where durable housing correlated with an 18% reduction in U5MR (OR: 0.82, 95% CI: 0.76–0.88) [20]. Conversely, in urban settings, durable housing increased child mortality risk by 2% (OR: 1.24, 95% CI: 1.04–1.49), perhaps reflecting other adverse factors in better-built high-risk areas. Similarly, Van de Poel and colleagues identified higher U5MR in premises with unfinished floors in urban areas (*β*: 0.13, *p* < 0.01), compared to no significant mortality effect in rural areas, where most dwellings have unfinished flooring [19]. This possibly represents slum dwellings and poor public health conditions.

#### Healthcare-Related Factors

Some authors explored access to and use of healthcare as well as health behaviors to construct urbanicity and rurality. Healthcare-related factors explored across this review include child immunization coverage, travel time and distance to the nearest health facility, ANC visits, birth by caesarean section, use of SBA, and facility delivery.

Vaccination coverage is important in explaining child mortality disparities, with countries exhibiting high levels of child vaccination coverage displaying more rapid declines in U5MR [21]. Across 23 SSA countries, urban children show higher full immunization coverage (FIC) (52.8%) than rural (40.7%, *p* < 0.001), often linked to socioeconomic disparities [24]. Richest wealth status was the most significant contributor (35.7%) to the urban–rural gap (*p* < 0.001), with 8% of the gap explained by distance to health facilities (*p* < 0.001) [24]. Obanewa and Newell echoed these findings in Nigeria, showing higher FIC in urban areas (69% higher in urban formal settings and 45% higher in slums) [25]. Place of delivery, maternal ANC attendance, and maternal education were significant across all settings, with maternal ANC attendance demonstrating the largest effect in rural areas (Rural: OR 8.37, 95% CI 5.34–13.12; Urban: OR 6.82, 95% CI 2.29–20.34; Slum: OR 8.07, 95% CI 2.15–30.25). In urban areas, maternal education had the largest effect (Rural: OR 4.99, 95% CI 2.48–10.06; Urban: OR 9.18, 95% CI 3.05–27.64; Slum: OR 5.03, 95% CI 1.52–16.65) [25]. Across 22 SSA countries, urbanization and urban population growth correlated with reduced child FIC (*r* = − 0.57, *p* = 0.01) [21]. Considering intra-urban inequities in FIC revealed that the general decline in FIC was more pronounced in urban slums (− 13%) than in urban areas as a whole (− 9%) in Zambia [21]. The same is exhibited in Kenya, whereby urban slum children have the lowest FIC rates in urban areas (82%) respective to rural areas (42%) [18]. Across both urban and rural areas, socioeconomic status was found to be positively correlated with facility delivery, with 90% and 33% in the highest and lowest social quintiles respectively delivering in a healthcare facility. This has also been exhibited in Tanzania (Rural: 54.4%, Urban: 88.3%) [26] and Ghana (adjusted OR: 1.59, 95% CI: 1. 07–2.37, *p* = 0.02) [27]. The determinants of using SBA were similar but of different strengths in rural and urban areas. Residing less than 4 km from a health facility was the greatest independent contributor to the variance in SBA in the urban areas (Rural: OR 6.05 95% CI 2.54–14.39, *p* < 0.001; Urban: OR 9.62, 95% CI 3.78–24.49, *p* < 0.001), whereas in rural areas, frequency of ANC attendance was the greatest independent contributor (Rural: OR 34.86, 95% CI 12.74–95.38, *p* < 0.001; Urban: OR 3.93, 95% CI 2.21–6.86, *p* < 0.001). Maternal education was more significant in rural areas, of 37.6% increased SBA use compared to 5% in urban areas (*p* = 0.001) [27].

Attendance of less than the WHO-recommended four ANC visits has been associated with higher NMRs across SSA, and thus is used as a proxy for exploring child mortality (28; 29). There is a consistently higher coverage of ANC in urban respective to rural areas. Adewuyi and colleagues found that in Nigeria, more rural women (61.1%, 95% CI: 58.5–63.5) underutilized ANC compared to their urban counterparts (22.4%, 95% CI: 20.0–25.1) [28]. Determinants were similar across urban and rural areas, but the strength of their effects differed. In rural areas, wealth index had a stronger effect (adjusted OR 2.17, 95% CI 1.68–2.81) than in urban areas (adjusted OR 2.05, 95% CI 1.51–2.79). Whereas in urban areas, lack of paternal education had the strongest effect (Rural: adjusted OR 2.03, 95% CI 1.72–2.43; Urban: adjusted OR 2.16, 95% CI 1.68–2.75).

Caesarean delivery has been correlated with increased NMRs across SSA [29] and is used as a proxy for child mortality in this review. The prevalence of caesarean delivery was higher in urban areas (10.37%, 95% CI: 8.99–11.75) than in rural areas (3.78%, 95% CI:3.17–4.39) across 28 SSA countries [30]. Approximately 81% of the rural–urban disparities in caesarean deliveries were attributable to the differences in child and maternal characteristics. Wealth index was the largest contributor to explaining the rural–urban disparity in caesarean deliveries. The likelihood of a caesarean section increased with wealth index in both urban (OR: 2.83, 95% CI: 2.11–3.80) and rural areas (OR = 2.58, 95% CI: 2.17–3.07). However, the odds were more significant in urban areas. Compared to women who had no ANC, those who had four or more ANC visits were more likely to deliver through caesarean delivery, with higher odds in rural areas (OR: 4.49, 95% CI: 3.42–5.89) compared to urban (OR: 2.71, 95% CI: 1.80–4.11).

Travel time to the nearest hospital is key in constructing urbanicity. In Tanzania, while core urban areas had the shortest average travel time to hospitals (4 min core urban; 41 min semi-urban; 89 min rural), NMRs were paradoxically higher in core urban areas (39.8/1000 live births, 95% CI: 26.3–59.9), compared to semi-urban (24.8/1000 live births, 95% CI: 19.6–31.4) and rural (21.9/1000 live births, 95% CI:16.8–28.5) areas, which displayed similar NMRs (*p* = 0.03) [4]. This aligns with Yelverton and colleagues who observed urban children lived closer to health facilities (1.9 km) than their rural (5 km) counterparts (*p* < 0.001) [31]. Lungu and colleagues found that in Malawi, even in the context of geographical proximity to healthcare services, those in urban slums may not seek healthcare, let alone of a timely nature; 61% of caregivers sought healthcare, while 53% sought it late [7].

#### Sociodemographic Factors

Sociodemographic factors are difficult to disentangle from other child mortality indicators outlines as they are often confounders or effect modifiers, rendering their independent influence on urbanicity and child mortality complex to assess. However, some papers included in this review examine their effects independently, specifically socioeconomic status (SES) and maternal education, revealing nuanced insights. SES emerges as the most significant determinant in whether a country experiences an urban or rural penalty for child mortality [5, 21]. Despite maintaining more favorable sociodemographic profiles, NMRs paradoxically remained highest in urban areas (OR: 1.94, *p* = 0.006) [8, 31]. In Zambia, urban poor children were 46% more likely to die than their poorest rural counterparts, suggesting that other unfavorable circumstances in urban settings offset socioeconomic advantage, highlighting the challenges in isolating its impact on child mortality along the urban–rural continuum [21]. Maternal education was significant in reducing child mortality across urban and rural areas; however, the effects of this were most pronounced in rural areas (Rural: OR 0.17, Urban: OR 0.06) [19]. Gruebner and colleagues identified a 22% reduction in the risk of infant death for educated mothers in urban areas (OR: 0.84, 95% CI: 0.73–0.96) compared to rural (OR:0.78) in Kenya, highlighting the larger effect in rural areas [20].

#### Child Disease and Morbidity Indicators

Child morbidity indicators and disease incidence demonstrating urban–rural disparities that are used to construct urbanicity include infections (diarrhoeal diseases and ARI) and childhood malnutrition and stunting. When considering urban and rural areas as dichotomous, there are conflicting findings regarding which has highest rates of infection and likelihood to seek and receive treatment. Ekholuenetale and colleagues found a higher prevalence of ARI among urban residents across 23 SSA countries, coupled with an increased likelihood to seek and receive treatment [32]. In Malawi, Lungu and colleagues had consistent findings of a declining urban advantage with respect to diarrhoeal and ARI rates yet established an urban penalty for access to and use of treatment services (Urban: 59.1%, Rural: 67.7%) [7]. Kimani-Murage and colleagues also found that those in rural areas were more likely to seek care for childhood ARIs respective to their urban counterparts (OR:1.4) [12]. However, when intra-urban inequities are accounted for there are more consistent findings. Mberu and colleagues found that slum children in Nairobi fared worse than children in rural areas of Kenya across child morbidity and health service indicators, with urban slums exhibiting a U5MR of 3.6 times higher than the rest of Nairobi as a whole [33]. Slum children are more likely to have illnesses such as diarrhea and ARI (Rural: 9.1%, Urban: 7.3% Slum: 24.6%) and simultaneously less likely to receive treatment (Rural: 58.1%, Urban: 56.7%, Slum: 42.7%) compared to their rural and urban counterparts. Shon echoes these findings whereby across 26 SSA countries, children in deprived urban settlements are 24% (OR = 1.24; CI 1.14–1.34) more likely to experience diarrhea compared to children in rural villages (OR = 0.96; CI 0.92–1) [22].

There are cross-country disparities in child stunting and malnutrition across urban and rural settings. In Tanzania, more children are stunted in rural areas (45%) compared to urban areas (35%), and underweight (Rural: 17%, Urban: 11%), with the urban–rural disparity widening due to slower rural decline (18; 26). Children from households in the lowest wealth quintiles accounted for a larger proportion of stunted children across both urban and rural areas. No interaction effect existed between residence and other determinants, and the urban–rural disparity was mainly caused by the discrepancy of the individual and household-level factors between rural and urban households [26].

## Discussion

This scoping review explored the narrowing urban advantage in child mortality in SSA by addressing two objectives: how urban and rural settings are operationalized, and, which social determinants related to urbanicity and rurality explain child mortality in these settings**.** This review highlights a persistent limitation in child health literature: the inadequate conceptualization and inconsistent classification of urban and rural environments. Most studies operationalized these settings as binary, relying on outdated national administrative definitions, obscuring diversity within and between urban and rural environments. Peri-urban, suburban, and informal urban areas are frequently misclassified or excluded, masking intra-urban disparities. These limitations hinder cross-study comparability and contribute to inconsistent findings in child mortality. However, some studies employed more nuanced methods, treating urbanicity as a continuum or stratifying urban areas into formal and informal zones, revealing more consistent patterns and highlighting the growing health burden in informal urban settlements. These findings underscore the need for refined spatial frameworks that better reflect the heterogeneity of contemporary urban and rural environments in SSA.

The review found no determinant of child mortality exclusive to either urban or rural settings. Environmental, healthcare-related, sociodemographic, and disease/morbidity-related factors were significant across all contexts, though their distribution and impact varied. Maternal education and household wealth were consistently protective, often more impactful in rural areas, likely reflecting lower baseline service access. The variation in the strength and distribution of determinants may be attributable to varying operationalizations of “urban” and “rural”, limiting the comparability of findings. Nonetheless, studies consistently show that intra-urban inequalities in these determinants are widening, sometimes exceeding urban–rural gaps. This underscores the need to look beyond binary classifications to address the complex spatial and social dynamics shaping child mortality across SSA.

### Reflections

#### Urbanicity as Binary

Most studies operationalized urbanicity and rurality as binary, misrepresenting the complexity of these settings. Findings were inconsistent, with some reporting a persistent urban advantage, others an emerging urban penalty, and some showing no clear pattern. This suggests the binary classification inadequately captures the structural and social determinants shaping child mortality across diverse contexts. Studies defined urban by population size, with thresholds ranging from 5000 to 20,000 inhabitants. These definitions, often drawn from outdated census data, lack standardized criteria, and are infrequently revised or re-evaluated. They are shaped by political influences tied to funding, infrastructure, and resource allocation [4]. While administratively convenient, this approach oversimplifies the heterogeneous realities of urban and rural settlements, leading to several limitations. First, it obscures the heterogeneity within urban and rural contexts, masking significant intra-urban disparities. Second, it overlooks transitional zones along the urban–rural continuum, such as peri-urban, suburban, and informal settlements. Data limitations further compound these challenges, with cross-sectional DHS data limiting causal inference. Legal and spatial ambiguity renders transient or unregistered populations uncaptured in informal urban areas, while rural estimates may be skewed by under-reporting due to weak infrastructure and surveillance. These oversights contribute to inconsistencies in findings, particularly when exploring whether an urban advantage or penalty exists in child mortality outcomes.

#### Urbanicity as Heterogenous

A smaller subset of studies considered urbanicity as heterogenous by employing more refined spatial measures, either by stratifying urban settings or treating urbanicity as a continuum. In these studies, a consistent trend emerged: children living in informal urban settlements fared worse than their formal urban, and occasionally rural counterparts. Determinants such as insecure tenure, overcrowding, inadequate sanitation, low maternal education, lack of health insurance, and exposure to environmental risk were common. Such consistent findings reflect not only improved methodological sensitivity, but also substantive changes in what constitutes ‘urban’. The expansion of informal settlements on a background of rapidly urbanizing regions has reshaped the urban health landscape. Increasing urban poverty, informal housing, and precarious livelihoods underscore how contemporary urban environments blur the traditional urban–rural divide. This convergence of evolving social determinants and outdated spatial classifications have made comparisons across studies more difficult, yet the consistency of findings in those that stratified or disaggregated urbanicity strengthens confidence in the observed trends. Studies using continuous measures of urbanicity offered additional nuance, revealing patterns missed by binary comparisons. While greater urbanicity generally correlated with lower U5MRs, this trend does not hold in deprived urban environments. Children residing in both rural and deprived urban settlements faced the greatest health burdens, indicating that location alone does not sufficiently predict outcomes. This highlights the importance of disaggregating urban areas, as treating urban as a monolith ignores heterogeneity across informal, peri-urban, and formal urban zones. Using continuous or spatially refined urbanicity measures offer a partial remedy to data quality issues by incorporating satellite imagery, despite remaining constrained by the limited health data in informal and remote areas. Considering urbanicity as heterogenous better captures how child health outcomes are shaped, offering clearer insight into the erosion of the urban advantage.

### Strengths and Limitations

A strength of this scoping review is the vast array of studies screened systematically, providing a comprehensive overview of the literature. Broad and flexible inclusion criteria maximized eligible studies for review and facilitated extensive results. Adherence to PRISMA-ScR guidelines ensured robustness of this study. The stratified synthesis by urbanicity type provides conceptual clarity and enhances interpretability. Despite notable strengths, limitations include language restriction to English, reliance on only three databases, and exclusion of grey literature, which may have overlooked eligible studies. Additionally, the use of binary DHS limited the exploration of intra-urban inequities. Analyses using continuous measures of urbanicity would better address child mortality determinants.

### Recommendations

Future research must move beyond binary definitions and adopt more nuanced, multidimensional constructions of urbanicity and rurality. Current literature fails to fully capture the complexity of these settings and the multitude of social determinants that prevail across these settings to shape child health outcomes. Defining urbanicity solely by population density is insufficient and fails to capture the heterogeneity of these environments. Urbanicity scales, such as those constructed by Dahly and Adair in the Philippines, offer a promising model for future studies to disentangle urban–rural child mortality differentials [34]. Alongside population density, they considered access to media, transportation, education, healthcare, and economic opportunity, at both household and community levels. This scale identified differences in urbanicity which were not previously apparent in binary models, enabling more robust illustrations of the relationship between urbanization and health [34]. However, limited data on slums and informal settlements, renders implementing urbanicity scales challenging. Addressing this gap requires in-depth qualitative insights into individual, household, and community variables prevailing in these settings, amid rapid urbanization and population growth. Promising approaches, such as the Nairobi Urban Health and Demographic Surveillance System (NUHDSS) highlight the potential for similar surveys in SSA to generate data for investigating intra-urban child health inequities [35]. Employing an urban continuum in demographic research, while necessary, poses financial and logistical challenges. Reclassifying urban and rural areas is politically controversial and impacts resource allocation [4]. In resource-deprived settings, prioritizing long-term research investments may be impractical or beyond capacity. Short-term micro-level solutions may be more realistic and politically less complicated [36]. Low-resource strategies such as stratifying pre-existing DHS data using satellite imaging have shown promise in more accurately characterizing urbanicity compared to DHS data alone [23].

This review provides the foundation for future research to undertake more nuanced approaches in examining child mortality differentials across urban and rural settings. Future studies must first acknowledge how urban areas have evolved over time—marked by rapid urbanization, insufficient infrastructure, and the expansion of informal settlements—and second, recognize urbanicity as a heterogeneous condition. This shift is essential to uncovering intra-urban inequities often obscured by binary classifications, informing policies, and interventions to address these disparities. Policy and interventions should account for the health consequences of the evolving urban landscape across the urban continuum, centered around improving child health across all settings while bolstering the most disadvantaged. Ultimately, addressing child mortality disparities in SSA requires analytic frameworks and policy responses that fully account for the spatial and social complexities of urbanization, thereby enabling the development of more equitable and contextually appropriate interventions.

## Conclusion

The erosion of the urban advantage in child mortality across SSA cannot be fully understood through traditional binary frameworks. The drivers of child health are not inherently urban or rural but are instead shaped by the distribution of key social determinants within and across settings. The growing prevalence of intra-urban inequities, particularly in informal settlements, has reconfigured the geography of child mortality risk. As urbanization continues to reshape population health landscapes, demographic and epidemiological research must adopt more flexible, context-sensitive conceptualizations of urbanicity. The use of stratified and continuum-based approaches to urbanicity will be essential for effective, equity-driven child health policies.

## Supplementary Information

Below is the link to the electronic supplementary material.

Supplementary File 1 (DOCX 57.6 KB)

## Data Availability

Data sharing is not applicable to this article as no new data were created or analyzed in this study.

## Appendix Summary of findings across the four key categories (environmental, healthcare-related, sociodemographic and disease/morbidity related) and their effect on the urban-rural gap

**Table:**

Dimension	Factor	No. of articles mentioning	Direction of association (U5MR)	Effect on urban-rural gap
Environmental	Electricity	2	Protective – reduces infant mortality	Widens gap – better access in urban areas
	Clean water and sanitation	4	Protective – linked to child survival	Widens gap – access deteriorates with urban growth. Urban areas show better access to safe drinking water and sanitation, providing a protective effect on child health, but rapid urban growth may undermine these benefits in slum areas
	Housing quality (durable, presence of a floor)	3	Mixed – protective in rural; higher risk in urban/slums	Widens gap. Urban slums show the highest risk, with better-built urban areas also showing increased mortality risk, possibly due to other urban factors. Rural areas with durable housing show lower mortality risk.
Healthcare related	Immunisation coverage	5	Protective – higher coverage = lower mortality	Urban slums show the greatest disadvantage, with vaccination rates lower than both formal urban and rural counterparts. Socioeconomic disparities continue to drive the urban–rural gap, though urbanisation may reduce coverage despite proximity to health services
	Travel time / distance to nearest healthcare facility	3	Shorter distance linked to higher SBA and lower mortality	Proximity does not equate to access or timely use, particularly in urban slums. Highlights a hidden urban penalty and suggests that physical access alone is insufficient in urban contexts.
	ANC	5	Protective – especially in rural areas	Urban areas show better ANC coverage, but socioeconomic factors (wealth, paternal education) have more pronounced effects in rural areas**.** Wealth plays a stronger role in rural settings, while paternal education is a key determinant in urban areas.
	Birth by caesarean section	3	Correlated with higher neonatal mortality	Urban areas have a higher prevalence of caesarean sections**,** but the disparity is driven by wealth rather than place of residence. Rural areas show stronger effect of ANC visits on caesarean delivery, potentially indicating access barriers in urban settings despite higher prevalence.
	SBA	4	Strongly protective across settings	Urban areas generally have higher SBA rates, but distance to facility and socioeconomic status are key factors. Maternal education and ANC attendance are more impactful in rural areas, but access barriers in urban slums may negate urban advantage.
Sociodemographic	SES	4	Strong predictor of healthcare use and child survival	Urban advantage is undermined among the poorest urban residents. SES disparities magnify intra-urban inequalities, leading to potential urban penalties.
	Maternal education	4	Protective – stronger in rural areas	Narrowing – Protective effect exists across the continuum, but greater relative benefit in rural areas, helping to narrow the urban–rural gap where educational attainment improves.
Disease and morbidity related	Infections (ARI and diarrhoeal)	5	Harmful – linked to poor access and late treatment	Mixed when urban/rural are compared in aggregate, but more consistent when intra-urban inequality is considered: **-** Urban advantage narrows or reverses **-** Slum children exhibit an urban penalty**,** faring worse than both rural and formal urban children.
	Childhood malnutrition and stunting	3	Harmful – driven by household-level deprivation	Urban advantage remains overall, but gap widening due to slower rural decline in malnutrition
