# Supplementary material for: Exploring the Factors Underlying the Narrowing Urban Advantage in Child Mortality in Sub-Saharan Africa: A Scoping Review
Source: J Urban Health. 2025 Sep 4;102(4):775–88. doi: 10.1007/s11524-025-00989-6 (PMC12484451; doi:10.1007/s11524-025-00989-6)
Supplement: Supplementary file 1 — (DOCX 57.6 KB) [file 11524_2025_989_MOESM1_ESM.docx]

# **Appendices**

## Appendix A: Final search strings

1. **PubMed**

("urban health"[MeSH Terms] OR "urban population"[MeSH Terms] OR "rural population"[MeSH Terms] OR "rural health"[MeSH Terms] OR "rural health services"[MeSH Terms] OR "Urban Health Services"[MeSH Terms] OR "suburban Health Services"[MeSH Terms] OR "urban*"[Title/Abstract] OR "rural"[Title/Abstract]) AND ("health status disparities"[MeSH Terms] OR "inequality*"[Title/Abstract] OR "disparit*"[Title/Abstract] OR "advantage*"[Title/Abstract] OR "disadvantage*"[Title/Abstract] OR "slum*"[Title/Abstract]) AND ("Africa South of the Sahara"[MeSH Terms] OR "Nigeria"[Title/Abstract] OR "Ghana"[Title/Abstract] OR "Tanzania"[Title/Abstract] OR "Kenya"[Title/Abstract] OR "Malawi"[Title/Abstract] OR "SSA"[Title/Abstract] OR "Sub-Saharan Africa"[Title/Abstract] OR "Liberia"[Title/Abstract] OR "Burundi"[Title/Abstract] OR "Ethiopia"[Title/Abstract] OR "Democratic Republic of the Congo"[Title/Abstract] OR "Niger"[Title/Abstract] OR "Eritrea"[Title/Abstract] OR "Guinea"[Title/Abstract] OR "Namibia"[Title/Abstract] OR "Togo"[Title/Abstract] OR "Sao Tome and Principe"[Title/Abstract] OR "Benin"[Title/Abstract] OR "Comoros"[Title/Abstract] OR "Eswatini"[Title/Abstract] OR "South Sudan"[Title/Abstract] OR "Republic of the Congo"[Title/Abstract] OR "Angola"[Title/Abstract] OR "Uganda"[Title/Abstract] OR "Rwanda"[Title/Abstract] OR "Guinea-Bissau"[Title/Abstract] OR "Zimbabwe"[Title/Abstract] OR "Equatorial Guinea"[Title/Abstract] OR "Mali"[Title/Abstract] OR "Madagascar"[Title/Abstract] OR "Mauritania"[Title/Abstract] OR "Sierra Leone"[Title/Abstract] OR "Zambia"[Title/Abstract] OR "Somalia"[Title/Abstract] OR "Botswana"[Title/Abstract] OR "Cabo Verde"[Title/Abstract] OR "Cote d'Ivoire"[Title/Abstract] OR "Seychelles"[Title/Abstract] OR "Cameroon"[Title/Abstract] OR "South Africa"[Title/Abstract] OR "Lesotho"[Title/Abstract] OR "Sudan"[Title/Abstract] OR "Mozambique"[Title/Abstract] OR "Senegal"[Title/Abstract] OR "The Gambia"[Title/Abstract] OR "Chad"[Title/Abstract] OR "Burkina Faso"[Title/Abstract] OR "Central Africa Republic"[Title/Abstract] OR "Mauritius"[Title/Abstract] OR "Djibouti"[Title/Abstract]) AND ("Child Mortality"[MeSH Terms] OR "infant mortality"[MeSH Terms] OR ("child"[MeSH Terms] OR "child*"[Title/Abstract] OR "infant*"[Title/Abstract] OR "neonat*"[Title/Abstract] OR "under-five"[Title/Abstract])) AND ("mortality"[Title/Abstract] OR "death*"[Title/Abstract])

1. **Embase**

(exp urban area/ or urban health/ or urban population/ or rural population/ or rural health care/ or rural health/ or (rural* or urban*).ti,ab,kf) and (exp health disparity/ or (inequality* or disparit* or advantage* or disadvantage* or slum*).ti,ab,kf) AND (exp childhood mortality/ or infant mortality/ or child death/ or child health/ or (exp child/ or (child* or infant* or neonat* or under-five).ti,ab,kf) and (mortality or death*).ti,ab,kf) AND (exp 'Africa south of the Sahara'/ or (Nigeria or Ghana or Tanzania or Kenya or Malawi or SSA or 'Sub-Saharan Africa' or Liberia or Burundi or Niger or Eritrea or Guinea or Namibia or Togo or 'Sao Tome and Principe' or Benin or Comoros or Eswatini or South Sudan or 'republic of the congo' or angola or Uganda or Rwanda or Guinnea-Bissau or Zimbabwe or 'Equatorial Guinea' or Mali or Madagascar or Mauritania or 'Sierra Leone' or Zambia or Somalia or Botswana or 'Cabo Verde' or 'Cote d'Ivoire' or Seychelles or Cameroon or 'South Africa' or Lesotho or Sudan or Mozambique or Senegal or 'The Gambia' or Chad or 'Burkina Faso' or 'Central Africa Republic' or Mauritius or Djibouti).ti,ab,kf)

1. **WOS**

TS=((urban* OR rural*) AND (inequality* OR disparit* OR advantage* OR disadvantage* OR slum*) AND (child* OR infant* OR neonat* OR "under-five") AND (mortality OR death) AND ("Africa south of the sahara" OR Nigeria OR Ghana OR Tanzania OR Kenya OR Malawi OR "Sub-Saharan Africa" OR Liberia OR Burundi OR Niger OR Eritrea OR Guinea OR Namibia OR Togo OR "Sao Tome and Principe" OR Benin OR Comoros OR Eswatini OR "South Sudan" OR "Republic of the Congo" OR Angola OR Uganda OR Rwanda OR "Guinea-Bissau" OR Zimbabwe OR "Equatorial Guinea" OR Mali OR Madagascar OR Mauritania OR "Sierra Leone" OR Zambia OR Somalia OR "Botswana" OR "Cabo Verde" OR "Cote d’Ivoire" OR Seychelles OR Cameroon OR "South Africa" OR Lesotho OR Sudan OR Mozambique OR Senegal OR "The Gambia" OR Chad OR "Burkina Faso" OR "Central African Republic" OR Mauritius OR Djibouti))

Appendix B: PRISMA-ScR (Preferred Reporting Items for Systematic Reviews and Meta-Analyses extension for Scoping Reviews) checklist.

| **SECTION** | **ITEM** | **PRISMA-ScR CHECKLIST ITEM** | **REPORTED ON PAGE #** |
| --- | --- | --- | --- |
| **TITLE** | | | |
| Title | 1 | Identify the report as a scoping review. | 1 |
| **ABSTRACT** | | | |
| Structured summary | 2 | Provide a structured summary that includes (as applicable): background, objectives, eligibility criteria, sources of evidence, charting methods, results, and conclusions that relate to the review questions and objectives. | 1 |
| **INTRODUCTION** | | | |
| Rationale | 3 | Describe the rationale for the review in the context of what is already known. Explain why the review questions/objectives lend themselves to a scoping review approach. | 2 |
| Objectives | 4 | Provide an explicit statement of the questions and objectives being addressed with reference to their key elements (e.g., population or participants, concepts, and context) or other relevant key elements used to conceptualize the review questions and/or objectives. | 2-3 |
| **METHODS** | | | |
| Protocol and registration | 5 | Indicate whether a review protocol exists; state if and where it can be accessed (e.g., a Web address); and if available, provide registration information, including the registration number. | Not Applicable |
| Eligibility criteria | 6 | Specify characteristics of the sources of evidence used as eligibility criteria (e.g., years considered, language, and publication status), and provide a rationale. | 4-5 |
| Information sources* | 7 | Describe all information sources in the search (e.g., databases with dates of coverage and contact with authors to identify additional sources), as well as the date the most recent search was executed. | 4 |
| Search | 8 | Present the full electronic search strategy for at least 1 database, including any limits used, such that it could be repeated. | Appendix A |
| Selection of sources of evidence† | 9 | State the process for selecting sources of evidence (i.e., screening and eligibility) included in the scoping review. | 4-5 |
| Data charting process‡ | 10 | Describe the methods of charting data from the included sources of evidence (e.g., calibrated forms or forms that have been tested by the team before their use, and whether data charting was done independently or in duplicate) and any processes for obtaining and confirming data from investigators. | 5 |
| Data items | 11 | List and define all variables for which data were sought and any assumptions and simplifications made. | 5 |
| Critical appraisal of individual sources of evidence | 12 | If done, provide a rationale for conducting a critical appraisal of included sources of evidence; describe the methods used and how this information was used in any data synthesis (if appropriate). | Not preformed |
| Synthesis of results | 13 | Describe the methods of handling and summarizing the data that were charted. | 5 |
| **RESULTS** | | | |
| Selection of sources of evidence | 14 | Give numbers of sources of evidence screened, assessed for eligibility, and included in the review, with reasons for exclusions at each stage, ideally using a flow diagram. | 5-6 |
| Characteristics of sources of evidence | 15 | For each source of evidence, present characteristics for which data were charted and provide the citations. | 6-7 + appendix D |
| Critical appraisal within sources of evidence | 16 | If done, present data on critical appraisal of included sources of evidence (see item 12). | Not preformed |
| Results of individual sources of evidence | 17 | For each included source of evidence, present the relevant data that were charted that relate to the review questions and objectives. | 7-13 + appendix D-E |
| Synthesis of results | 18 | Summarize and/or present the charting results as they relate to the review questions and objectives. | 7-13 + appendix D-E |
| **DISCUSSION** | | | |
| Summary of evidence | 19 | Summarize the main results (including an overview of concepts, themes, and types of evidence available), link to the review questions and objectives, and consider the relevance to key groups. | 14-16 |
| Limitations | 20 | Discuss the limitations of the scoping review process. | 16 |
| Conclusions | 21 | Provide a general interpretation of the results with respect to the review questions and objectives, as well as potential implications and/or next steps. | 14-17 |
| **FUNDING** | | | |
| Funding | 22 | Describe sources of funding for the included sources of evidence, as well as sources of funding for the scoping review. Describe the role of the funders of the scoping review. | 18 |

JBI = Joanna Briggs Institute; PRISMA-ScR = Preferred Reporting Items for Systematic reviews and Meta-Analyses extension for Scoping Reviews.

* Where *sources of evidence* (see second footnote) are compiled from, such as bibliographic databases, social media platforms, and Web sites.

† A more inclusive/heterogeneous term used to account for the different types of evidence or data sources (e.g., quantitative and/or qualitative research, expert opinion, and policy documents) that may be eligible in a scoping review as opposed to only studies. This is not to be confused with *information sources* (see first footnote).

‡ The frameworks by Arksey and O’Malley (6) and Levac and colleagues (7) and the JBI guidance (4, 5) refer to the process of data extraction in a scoping review as data charting*.*

§ The process of systematically examining research evidence to assess its validity, results, and relevance before using it to inform a decision. This term is used for items 12 and 19 instead of "risk of bias" (which is more applicable to systematic reviews of interventions) to include and acknowledge the various sources of evidence that may be used in a scoping review (e.g., quantitative and/or qualitative research, expert opinion, and policy document).

*From:* Tricco AC, Lillie E, Zarin W, O'Brien KK, Colquhoun H, Levac D, et al. PRISMA Extension for Scoping Reviews (PRISMAScR): Checklist and Explanation. Ann Intern Med. 2018;169:467–473. [doi: 10.7326/M18-0850](http://annals.org/aim/fullarticle/2700389/prisma-extension-scoping-reviews-prisma-scr-checklist-explanation).

Appendix C: Results Table

Three categories emerged based on how urbanicity was operationalised in the included studies: (1) binary urban-rural classification (appendix Ci), (2) stratified urban areas (formal, informal/slum, rural) (appendix Cii)), and (3) continuous urbanicity using spatial metrics or satellite imagery (appendix Ciii).

Ci. Urbanicity as binary dichotomy

| Urbanicity as a binary dichotomy (urban + rural) | | | | | | | |
| --- | --- | --- | --- | --- | --- | --- | --- |
| Reference | Study Design | Data source | Country | Factors considered across urban continuum to explain mortality | Evidence on underlying factors | Mortality/ morbidity outcomes | Direction / magnitude of change |
| Norris, M., Klabbers, G., Pembe, A. B., Hanson, C., Baker, U., Aung, K., Mmweteni, M., Mfaume, R. S., & Benova, L. (2022) | Cross-sectional | DHS (2000- 2015) | 21 SSA countries with a focus on Tanzania | Population size. Caesarean section, hospital births, SBA, maternal education, weighing at birth. | More births in urban were by caesarean section (1.9%) than rural (3.4%). More hospital births in urban (61.5%) than rural (20.7%). Higher SBA presence in urban (86.0%) than rural (51.8%). New-borns in urban more likely be weighed at birth; 87.9% of urban & 53.6% of in rural. Mothers in urban had a higher level of education (secondary or higher) at 29.7%, compared with 8.0% in rural. | Urban has higher odd of neonatal mortality (OR=1.94, p=0.006) compared with rural | Urban penalty |
| Yelverton, V., Hair, N. L., Ghosh, S. H., Mfinanga, S. G., Ngadaya, E., Baumgartner, J. N., Ostermann, J., & Vasudevan, L. (2022). | Cross-sectional | DHS 2015-16 | Tanzania | Population size. Maternal education, home delivery, household wealth, distance to health facility. | Rural–urban disparities in vaccination coverage and timeliness were observed for all vaccines. Among rural and urban children, lower maternal education (U= OR: 1.48, 95% CI: 1.12-1.97; R= 1.47, 95% CI: 1.25-1.73) and delivery at home (U= 0.20, 95% CI: 0.09-0.43; R= 0.48, 95% CI: 0.42-0.54) were associated with increased risk of delayed vaccination. In rural, less household wealth (−0.44 (SD = 0.58) and greater distance to a health facility (4.99 (SD= 5.45) associated with increased risk of delayed vaccination (wealth more strongly related in rural than urban (R= OR: 1.20, 95% CI: 1.11-1.31; U= OR: 1.10, 95% CI: 1.01-1.21). distance only significant in rural facility (R= OR: 0.96, 95%CI: 0.94-0.97; U= OR: 1, 95%CI: 0.97-1.03) Effect of delivery at home on the timeliness of vaccinations due at birth (BCG, OPV0) larger among urban children than rural. | For all vaccine doses, rates of total coverage, rates of documented vaccination coverage, rates of maternal vaccination recall, and rates of vaccination timeliness, higher among urban children than rural children. Rural children tended to experience greater delays than urban children (OR: 0.84, 95% CI: 0.76-0.94). | Urban advantage |
| Yaya, S., Uthman, O. A., Okonofua, F., & Bishwajit, G. (2019) | Cross-sectional | DHS 2016 | 35 SSA countries | Population size. Maternal age, education, use of media, wealth index, size of baby, age at 1^st^ birth | Significant urban-rural differentials in U5MR across bio-demographic, socioeconomic and proximate factors. Wealth index and media access were the most important factor in most countries. Child sex, household head’s sex, parity, and mother’s employment status did not sow any significant contribution to the inequality in any of the countries. | 16 countries showed statistically significant pro-rural inequality (U5MR higher in rural), 2 showed statistically significant pro-urban inequality (U5MR higher in urban) and remaining 17 countries showed no statistically significant inequality. | 16: urban advantage  2: urban penalty  17: no significant difference |
| Lungu, E. A., Biesma, R., Chirwa, M., & Darker, C. (2019) | Cross-sectional | DHS 1992-2015 & MICS (multiple indicatory cluster survey) 1995-2014. 13 different child health indicators used. | Malawi | Population size. 13 different child morbidity indicators- | Diarrhea and fever treatment services = declining urban advantage, now urban penalty. ITN use= urban advantage- continued increasing until 2006 then rapidly declined up to 2014 & increased again in 2016. Full immunization coverage for children at 12 = declining urban advantage, moving to urban penalty (RD = − 6) in 2010, almost equal utilization in 2014 (RD = 0.6) & a reversal to a slight urban advantage in 2016 (RD = 2.2). Prevalence of diarrhea low across the surveys but pattern of increase in urban advantage from 1992 to 2004 & like other child morbidity indicators showing a declining urban advantage through 2010 DHS= urban penalty followed by slight urban advantage (rate difference of less than 2%) in 2104 & reversal to urban by 4 percentage points) in 2016. Pattern of urban–rural differentials in underweight children = rural advantage from 1992 to 2004, then reversal to urban advantage in 2006, to equal burden and an urban advantage in 2015. | Except for underweight which has largely moved from a rural to an urban advantage, the rate differences for the rest of child morbidity indicators in 2015/2016 reflect lower levels in 2015 compared to 2000. | Mixed depending on morbidity indicator. |
| Levira, F., & Todd, G. (2017) | Cross-sectional | DHS (2009-10, census (2012) & household budget survey (2011-12) | Tanzania | Population size. Household wealth, childhood stunting, ITN use, FIC, ANC, facility delivery. | Low-wealth quintiles accounted for a larger proportion of stunted children. Children/pregnant women sleeping under ITN similar for urban and rural (77% for children, 79% for pregnant women)- but,access to curative treatment for malaria was higher for pregnant urban women. 78% urban vs. 60% rural took an antimalarial drug during their last recorded pregnancy. Full vaccination is lower in urban areas (69% urban vs. 85% rural) ​(Table6) -, BCG vaccinations were similar for urban and rural areas, but polio (84% urban vs. 97% rural), DPT (81% urban vs. 90% rural), and measles (80% urban vs. 94% rural) show disparities. Lowest wealth quintiles have lower vaccination coverage. Coverage, and use, of ANC was higher in urban. Facility delivery higher in urban (82 vs. 42% rural). Use of a facility for delivery higher among the highest social quintile compared to the lowest (90 vs. 33%). | More stunting in rural (45% vs. urban 35%) and underweight (17% vs. urban 11%) | Mixed depending on morbidity indicator. |
| Ekholuenetale, M., Nzoputam, C. I., Okonji, O. C., Barrow, A., Wegbom, A. I., & Edet, C. K. (2023) | Cross-sectional | DHS (2006-21), | 37 SSA countries | Population size. ARI. | Urban resident is associated with ^ likelihood of seeking treatment & receiving antibiotics | A higher prevalence of ARI was observed among urban residents. | Urban penalty for disease burden, but urban advantage for seeking treatment. |
| Ameyaw, E. K., Kareem, Y. O., Ahinkorah, B. O., Seidu, A. A., & Yaya, S. (2021) | Cross-sectional | DHS (2010-18) | 23 SSA countries | Population size, FIC, birth order, maternal age, education, wealth, distance to health facility, | 76.5% of rural p<0.001–urban variation in full immunisation was attributable to differences in child and maternal characteristics. (table 6).Household wealth was an important component contributing to the rural–urban gap. Specifically, richest wealth status substantially accounted for immunisation disparity (35.7%; p<0.001). First and sixth birth orders contributed 7.3% and 14.9%, respectively, towards the disparity while 7.9% of the disparity was attributable to distance to health facility (p<0.001). | More children in urban fully immunised (52.8%) than rural (40.7%). | Urban advantage |
| Van De Poel, E., O'Donnell, O., & Van Doorslaer, E. (2009) | Cross-sectional | DHS- 1995-2001 | Benin, Central African Republic, Chad, Guinea, Mali, and Niger | Population size, SES, toilet, water, electricity, health facilities, public transport, maternal education | Rural penalty for child mortality- most important contributory variable = disadvantageous environmental conditions such as limited electricity, quality housing materials and water supply. In rural- the few households with an electricity supply = significantly reduced probability of infant death (0.13, p<0.01). In urban = mortality risk higher among households living in premises with no finished floor (0.128, p<0.01)- identifies slum dwellings & poor public health conditions found there. In rural = majority of dwellings have no finished floor & not significantly with mortality risk. Protective effect of maternal primary education for child mortality more significant in rural areas (R= 0.167, U=0.059)- but there is no significant difference between urban and rural areas for this factor | Rural penalty for child mortality | Urban advantage |
| Zhu, W. J., Zhu, S., Sunguya, B. F., & Huang, J. Y. (2021) | Cross-sectional | DHS 1991-2016 | Tanzania | Population size, age at birth, education levels, BMI of mother, sanitation, facility birth. | Factors associated with child stunting in urban: Individual-level= lower stunting odds among children who were younger, female (odds ratio (OR): 0.63, 95% CI: 0.55–0.72, p < 0.001), born in medical institutions (OR: 0.61, 95% CI: 0.45–0.83, p = 0.002), had normal or high birth weight (OR: 0.46, 95% CI: 0.39–0.55, p < 0.001), and had a shorter duration of breastfeeding. Household-level, = lower stunting odds in children whose mother had fewer children, was obese, and attended a higher level of education. Compared with non-working mothers, children with farmer mothers had higher odds of stunting (OR: 1.34, 95% CI: 1.12–1.60, p = 0.003). Children born in households whose heads were male had lower chances of stunting (OR: 0.65, 95% CI: 0.55–0.76, p < 0.001), and the type of toilet was significantly associated with childhood stunting, as children from the family who had worsened sanitation (pit latrine, no facility/bush/field) were more likely to become stunted.  Rural= except for the total number of children, the highest education level of the mother, and the source of drinking water, the determinants of child stunting were similar to urban children. Compared to underweight mothers, overweight and obese mothers halved the odds of child stunting. Children whose family used pit latrine were associated with a 129% increase in the odds of stunting compared to children whose family used the flush toilet. These three predictors showed a greater influence on rural children than on urban children. | Total stunting prevalence declining in Tanzania, but U-R disparity widened as the decline was slower in rural.  No interaction effect existed between residence and other determinants; U-R disparity mainly caused by discrepancy of the individual-level and household-level factors between rural and urban households. | Urban advantage, but more rapid rates of improvement in urban areas. |
| Adewuyi, E. O., Auta, A., Khanal, V., Bamidele, O. D., Akuoko, C. P., Adefemi, K., Tapshak, S. J., & Zhao, Y. (2018) | Cross-sectional | DHS 2013 | Nigeria | Population size, health knowledge, household assets, maternal age, education, autonomy | Factors associated with greater odds of ANC underuse in rural = maternal non-working status (AOR: 1.27; 95% CI: 1.11, 1.45), birth interval < 24 months (AOR: 1.26; 95% CI: 1.09, 1.46), single birth type (AOR: 1.46; 95%CI: 1.00, 2.15), not listening to radio at all, lack of companionship to health facility and not getting money for health services. In both rural and urban residence, lack of maternal education (R: AOR: 1.93; 95% CI: 1.11, 1.45 & U= AOR: 1.44; 95% CI: 1.10, 1.87) ,lack of husband’s education (R: AOR: 2.03; 95% CI: 1.72, 2.43 & U: AOR: 2.16; 95% CI: 1.68, 2.75), wealth index/living in poorer households (R: AOR: 2.17; 95% CI: 1.68, 2.81 & U: AOR: 2.05; 95% CI: 1.51, 2.79), maternal age younger than 35 (R: AOR: 1.16, 95%CI: 1.04, 1.31 & U= AOR: 1.25; 95% CI: 1.03, 1.49), frequency of watching television, distance to- and permission to visit health facility were significantly associated with ANC underuse. In contrast to the rural residence, maternal working status, birth interval, birth type, access to radio, the problem of money and being accompanied to a health facility were not significantly associated with underutilization of ANC in urban Nigeria. In urban residence, mothers professing Islam (AOR: 1.64; 95%CI: 1.21, 2.26 compared to Christian), those who did not read newspaper at all (AOR: 1.74; 95%CI: 1.17, 2.59), and those who lacked health insurance (AOR: 3.41; 95%CI: 1.53, 7.58), had greater odds of ANC underuse. (These factors did not apply in rural areas) | More rural women (61.1%; 95% CI: 58.5–63.5) underutilized ANC compared to urban (22.4; 95% CI: 20.0–25.1). | Urban advantage |
| Ahinkorah, B. O., Aboagye, R. G., Seidu, A. A., Okyere, J., Mohammed, A., Chattu, V. K., Budu, E., Adoboi, F., & Yaya, S. (2022) | Cross-sectional | DHS 2010-20 | 28 SSA countries | Population size, caesarean delivery, maternal age, education, ANC, media exposure, wealth index | 81% of rural–urban disparities in caesarean deliveries attributable to the differences in child and maternal characteristics. Wealth index (39.2%), antenatal care attendance (13.4%), parity (12.8%), mother’s educational level (3.5%), and health insurance subscription (3.1%) explained 72% of the rural–urban disparities. Likelihood of caesarean section increased with wealth index in both urban ([aOR = 2.83; 95% CI = 2.11–3.80] and rural areas [aOR = 2.58; 95% CI = 2.17–3.07]). However, the odds were higher in urban areas. Compared to women who had no antenatal care, those who had 4+ ANC visits were more likely to deliver through caesarean delivery, with higher odds in rural [aOR = 4.49; 95% CI = 3.42–5.89] compared to urban [aOR = 2.71; 95% CI = 1.80–4.11]. Women with a higher level of education more likely to deliver through caesarean delivery than those with no formal education in both rural and urban areas-odds were significant in rural areas only [aOR = 1.42; 95% CI = 1.15–1.76]. Women covered by health insurance more likely to deliver through caesarean delivery than those who were not covered in rural and urban areas- odds higher in rural areas [aOR = 1.65; 95% CI = 1.41–1.94] when compared to urban [aOR = 1.56; 95% CI = 1.39–1.75] | Caesarean delivery higher in urban (10.37%; 95% CI = 8.99–11.75) than rural (3.78%; 95% CI = 3.17-4.39) across the 28 countries | Urban advantage |
| Saaka, M., & Akuamoah-Boateng, J. (2020) | Cross-sectional | Population & Housing Census (2010)- 720 post-partum women. | Ghana | Population size, SBA, maternal education, wealth, knowledge of danger signs, distance from health facility, ANC | Determinants in U&R similar but of diff levels & strengths. Rural: key determinants of SBA were maternal age (AT LEAST 35) (OR= 9.8 CI=2.21-43.06 at P=<0.01), high household wealth index (OR=2.37 CI=1.09-5.89 at p = <0.05), parity PARA > 1 (OR= 11.88 CI=3.76-37.56 at p=<0.001), SHORTER distance from health facility (OR=6.05 CI=2.54-14.39 at p=<0.001), HIGH decision-making autonomy of women (OR=8.24 CI=3.32-20.08 at p=<0.001), low perceived barriers to skilled birth services (OR= 10.98 CI=7.27-50.08 at p=<0.001), at least 4-frequency of antenatal care (ANC) attendance (OR=34.86 CI=12.74-95.38 at p=<0.001), and no knowledge of danger signs during delivery (OR=2.91 CI1.03-8.19 at p=<0.5). Urban: consistent determinants were parity p > 1 (OR= 4.2 CI=1.92-9.21 at p=<0.001), distance from health facility less than 4km (OR= 9.62 CI=3.78-24.49 at p=<0.001), higher women's autonomy in decision-making (OR=2.65 CI=1.41-4.97 at p=<0.01), low perceived barriers to skilled birth services (OR= 4.22 CI=2.32-7.49 at p=<0.001), and at least 4-frequency of ANC attendance (OR=3.93 CI=2.21-6.86 at p=<0.001). Household wealth index, maternal age, and knowledge of danger signs during delivery were therefore not important determinants of SBA in the urban areas. 37.4% of births in rural & 47.1% in urban among women with no education were assisted by SBAs, compared to 75% in rural & 52.5% in urban among those with secondary education or more (p= 0.001)- % difference between education levels was greater in rural (p=0.001). Distance to the nearest health facility had a significant influence on SBA- rural = 75.0% of women living within 1–3 km of a health facility gave birth with SBAs compared with 30.2% of women living at least 4 km from a facility (p= <0.001). SBA= 34.9 and 3.9 times higher for women who attended ANC at least four times in rural & urban areas, respectively, compared to those who attended ANC < 4. | SBA higher in urban than rural (AOR = 1.59; CI: 1. 07–2.37; p=0.02). | Urban advantage |

Cii: Urbanicity stratified by urban formal, urban informal/slum and rural

| Urbanicity stratified by urban formal + urban informal/slum + rural | | | | | | | |
| --- | --- | --- | --- | --- | --- | --- | --- |
| Reference | Study design | Data set | Country | Factors considered across urban continuum to explain mortality | Evidence on underlying factors | Mortality / morbidity outcomes | Direction / magnitude of change |
| Obanewa, O. A., & Newell, M. L. (2020) | Cross-sectional | DHS 2003-13 | Nigeria | Population size, improved water and sanitation, education, mothers age, household media exposure, distance to health facility, religion | In each setting, factors significantly associated with FIC were place of delivery (R= 1.47 (1.12,1.94); U= 2.62 (1.43,4.79) S= 5.39 (2.18,13.33), maternal ANC attendance (R= 8.37 (5.34,13.12) U= 6.82 (2.29,20.34) S=8.07 (2.15,30.25) and maternal (higher) education level = R= 4.99 (2.48,10.06) U= 9.18 (3.05,27.64) S= 5.03 (1.52,16.65). FIC significantly increased with (1) higher maternal education & (2) mothers age at birth in all areas- association largest in urban. Compared Muslim, a Christian = higher odds of FIC, especially in the slums. FIC was significantly associated with birth order in urban formal & slums, religion in rural & slums, maternal age in rural & urban formal, media exposure and distance to the health facility (1.76 (1.35,2.29) in rural, and with current maternal marital status and decision-maker of maternal income in slums. FIC was not significantly associated in any setting with sex of the household head, mother employment status and household wealth. The association between FIC and (1) household media exposure 1.91 (1.43,2.54), (2) distance to nearest health facility (1.76; 1.35-2.29) was significant only in rural areas. | FIC lowest in rural areas, with slum FIC coverage higher than in rural but lower than in urban formal areas (69% higher in urban formal, 45% higher in slum) FIC for rural, urban formal and slum rose from 7.4, 25.6 and 24.9% respectively in 2003 to 15.8, 45.5 and 38.5% in 2013, and varied across socio-demographics. *However, with the introduction of community variables and interaction terms in Model 3, FIC for an urban child (adjusted Odds Ratio (aOR) =1.60, 95% CI = 0.60–4.24) compared to the rural child remained higher, but was no longer statistically significant*. | Rural penalty. Urban slums fare worse than formal urban. |
| Fotso, J. C., Ezeh, A. C., Madise, N. J., & Ciera, J. (2007) | Cross-sectional | DHS 1990-2007 & Nairobi cross-sectional slum survey (NCSS) 2000 | 22 SSA countries with a focus on Kenya and Zambia | Population size, toilet facility, access to piped water, access to health services, FIC | More rapid urban population growth is associated with negative trend in access to safe drinking water (correlation of -0.42; p value of 0.07) and in vaccination coverage (correlation of -0.57, p value of 0.01), and ultimately to increasing child mortality. Intra-urban disparities in child health Kenya and Zambia for example.  Kenya: Access to clean water has been deteriorating in urban slums between 1993 (87%) and 2003 (54%)= drop of 33 %, compared with 18 percent in urban Kenya and 5 percent in rural areas. % of urban children who were fully immunized dropped from 76% in 1993 to 48% in 2003. Within slums, immunization rates were lower and dropped from 71% to 43%.  Zambia: 1992 and 1996 Zambian DHS- Reversal in household SES between the two surveys and lower utilization of health care. In 1992, 82% of households in slums had access to piped water, but this dropped to 68% in 1996. General decline in vaccination coverage between 1992 and 2001 more pronounced in the slums (-13%) than in urban Zambia as a whole (-9%). | Kenya: slum children exhibit higher and faster rates of infant mortality (39% increase from 1993-1998) than urban children (14%) and rural children (22%).  Zambia: 1992 and 1996 Zambian DHS- urban poor children = 46% higher probability of dying in infancy than the poorest rural children. Increase in infant mortality in slums of 7% vs 3% reduction in rural areas between the 2 DHS surveys. | Urban poor penalty |
| Gruebner, O., Lautenbach, S., Khan, M. M. H., Kipruto, S., Epprecht, M., & Galea, S. (2015) | Cross-sectional | Kenya Population & Housing Census (2009) | Kenya | Population size, structural quality of housing, access to water & sanitation. | Living in urban reduced health promoting effects of better structural quality of housing (i.e. poor or good versus non-durable). Durable housing quality in urban areas = risk factor for infant death compared to rural. Compared to non-durable structural quality of housing, poor housing in rural reduced infant death risk by 33% (OR 0.665, 95% CI 0.626–0.706), good housing reduced risk of infant death by 31% (OR 0.690, 95% CI 0.650–0.733), and durable housing reduced risk for infant death by 18% (OR 0.821, 95% CI 0.764–0.883). These factors were amplified in urban areas, by 1.243 (95% CI 1.029–1.509) for poor housing, by 1.329 (95% CI 1.119–1.589) for good housing, and by 1.242 (95% CI 1.044–1.488) for durable housing- they significantly reduced infant death risk in these areas by 17% (in the case of poor housing) and 8% (good housing). But durable housing in urban areas increased infant death risk by 2%. For urban areas, effect of secondary education level was 0.837 (95% CI 0.731–0.959) times the OR in rural areas (0.782), which = a 22% reduction in risk for infant death for better educated mothers in urban areas. | higher risk of death in urban non-slum (OR 1.121, 95% CI 1.082–1.161) or slum areas (OR 1.487, 95% CI 1.407–1.570) compared to rural areas | Urban penalty |
| Kimani-Murage, E. W., Fotso, J. C., Egondi, T., Abuya, B., Elungata, P., Ziraba, A. K., Kabiru, C. W., & Madise, N. (2014) | Longitudinal | DHS (1993-2008) & Nairobi Urban Health and Demographic Surveillance System (NUHDSS) 2003-10 | Kenya | Population size, adolescent childbearing, duration of exclusive breastfeeding, FIC, diarrhoea, health seeking for illness. | Reversal in the ratio between urban and rural of determinants of child mortality including adolescent childbearing (0.8–1.1), median duration of exclusive breastfeeding (1.0–0.5), child immunization (1.0–0.9), diarrhoea morbidity (0.9–1.1) and health seeking for childhood illnesses (acute respiratory infections) (1.4–0.9), all in favour of rural areas. | Downward trend in IMR, CMR and U5MR in both rural and urban areas. Decline was more rapid and statistically significant in rural areas but not in urban areas, hence the gap in urban–rural differentials narrowed over time. U5MR was very high in the urban slums (104) compared with 75, 72.5 and 73 for the urban as whole, rural and Kenya as a whole, respectively. | Urban slum penalty |
| Mberu, B. U., Haregu, T. N., Kyobutungi, C., & Ezeh, A. C. (2016) | Comparative analysis of cross-sectional data | DHS (2003-14) & Kenya National census 1999 & NCSS 2000-12 | Kenya | Child morbidity indicators= nutrition, FIC, breastfeeding, ARI, fever, diarrhoea, ANC, place of delivery, SBA. | All child morbidity and health service indicators worse for slum children in Nairobi than rural Kenya. Vaccination: slums= (45.2%) formal urban (83%), rural (77.4%) Childhood illnesses (diarrhoea, ARI) higher in slums than rural and urban areas. Treatment seeking for childhood illnesses was better in slums and urban than rural areas. Slum less likely to get treatment for diarrhoea (S= 42.7, R= 58.1, U=56.7), and less likely to be vaccinated (S= 45.2, R=77.4, U=83.0). The decline in the prevalence of childhood illnesses was faster in slums, whereas the increase in immunization coverage and treatment seeking behaviour was higher in rural Kenya. Slums had higher coverage of ANC (S= 96.2, R=94.0, U= 97.8), SBA (S=82.4, R=50.4, U-82.4), and institutional delivery (S=83.0, R= 49.5, U= 82.0) than rural areas. | U5MR in Nairobi slums was about 3.6 times higher than that of Nairobi as a whole. | Urban slum penalty |
| Gunther, I., & Harttgen, K. (2012). | Longitudinal | DHS (1998-2006) | SSA | Access to water and sanitation, overcrowding, housing quality, population size | Ethiopia in 2000, the child mortality rate is 51.5 deaths per 1000 in formal settlements and 125.9 deaths per 1000 in slums, resulting in a ratio of slum to formal settlement mortality  The higher mortality areas indicate that although living in slums might pose children than living in rural areas (as found in previous cess to basic health infrastructure still seems to be better thus offsetting the "morbidity penalty" of slums. | Difference in child mortality within urban > difference in child mortality between urban & rural. child mortality 65% higher in urban slum dwellings than in formal urban settings, whereas it is only 16% lower than in rural areas. -indicates that whilst living in slums poses higher child health risk, urban access to basic infrastructure still better than in rural. | Rural penalty, narrowing urban advantage and stark intra-urban inequities. |

Ciii. Urbanicity as continuous

| Urbanicity as continuous | | | | | | | |
| --- | --- | --- | --- | --- | --- | --- | --- |
| Reference | Study design | Data source | Country | Factors considered across urban continuum to explain mortality | Evidence on underlying factors | Mortality / morbidity outcomes | Direction of change |
| Corker, J. (2017) | Cross-sectional | DHS (2001-11), population census (2002-10) | Benin, Burkina Faso, Cote d’Ivoire, Ghana, Guinea, Mali, Niger, and Senegal | Urban amenities (electric, water, & sanitation) | Urban more likely than rural to have electrification (U= 74 %; R= 15.2%), access to clean water (U=82.2% & R= 51.7%) and improved sanitation (U=69.8%; R=20.2%). Proportional access to these urban amenities & U5MR declines as city size decreases. | Children in urban areas are 7% more likely to reach age five than their rural counterparts (significant at p<.01 for the log-rank test of equality). | Urban advantage |
| Macharia, P. M., Beova, L., Pinchoff, J., Semaan, A., Pembe, A. B., Christou, A., & Hanson, C. (2023). | Cross-sectional | DHS 2015-16 | Tanzania | Travel time & distance to nearest health facility | DHS rural and urban classes had an average of 14 and 78 min of travel time to the nearest hospital. In the three stratified urbanicity classes, average travel time was 89 min in rural, 41 min in semi-urban and 4 min in core urban. (Most semi-urban and core urban were within 30 min of the nearest public hospital). | Mortality estimates for rural areas did not differ between DHS and satellite classifications. NMR in semi-urban were similar to levels in rural categories of both DHS and satellite. Core urban = the highest NMR (39.8/1000 live births); significantly higher than semi-urban (24.8, 19.6-31.4) and rural (21.9, 16.8-28.5) NMRs (p=0.03). | Urban penalty (most marked in core urban areas) |
| **Shon H. (2024)** | Cross-sectional | DHS (2011-2019) and the Global Human Settlements Layer (2019) | 26 SSA countries | Population size. 4 different child morbidity indicators (fever, anaemia, diarrhoea, cough) | Deprived urban settlements exhibit resemblances to rural towns in economic status but shows lower scores in educational attainment and living standards.  Diarrhoea: children in deprived urban settlements are 24% (OR = 1.24; CI 1.14–1.34) more likely to experience diarrhoea compared to children in rural villages, whereas children in rural villages are 4% (OR = 0.96; CI 0.92–1) less likely to suffer from diarrhoea.  Cough: children living in rural clusters, rural towns, deprived urban settlements, urban clusters, and urban centers exhibit 7% (OR = 1.07; CI 1.02–1.12), 11% (OR = 1.11; CI 1.05–1.18), 22% (OR = 1.22; CI 1.13–1.32), 24% (OR = 1.24; CI 1.14–1.35), and 23% (OR = 1.23; CI 1.12–1.35) higher odds of experiencing cough, respectively, than children living in rural villages. | In comparison to children in rural villages, children in rural towns are 6% (OR = 0.94; CI 0.9–0.98), children in urban clusters with deprived settlements are 9% (OR = 0.91; CI 0.86–0.96), and children in urban centres with deprived settlements are 16% (OR = 0.84; CI 0.78–0.9) less likely to lose their lives. The results suggest that the settlement-type variable offers a more nuanced explanation of the spatial disparities in child mortality compared to the simplistic urban-rural dichotomy. Model 3 (stratified): children living in deprived urban settlements exhibit a weak urban advantage (OR = 0.94; CI 0.88–1.01). The odds ratios of the urban cluster (OR = 0.85; CI 0.8–0.91) and the urban center (OR = 0.83; CI 0.77–0.9) are lower than those of the urban cluster with deprived settlements and the urban center with deprived settlements in Model 2, respectively. Deprived urban settlements have a relatively high level of mortality within urban regions when compared to their urban counterparts. | Urban advantage |
